# Using the Deep Learning Algorithm to Determine the Presence of Sacroiliitis from Pelvic Radiographs

**DOI:** 10.3390/life15060876

**Published:** 2025-05-29

**Authors:** Ming Xing Wang, Jeoung Kun Kim, Donghwi Park, Min Cheol Chang

**Affiliations:** 1 College of Economics and Management, Wenzhou University of Technology, Wenzhou 325000, China; 20240095@wzut.edu.cn; 2 Department of Business Administration, School of Business, Yeungnam University, Gyeongsan-si 38541, Republic of Korea; kimjk70@yu.ac.kr; 3 Seoul Spine Rehabilitation Clinic, Ulsan-si 44607, Republic of Korea; bdome@hanmail.net; 4 Department of Rehabilitation Medicine, College of Medicine, Yeungnam University, Daegu 42415, Republic of Korea

**Keywords:** deep learning, convolutional neural network, sacroiliitis, radiograph, computed tomography, sacroiliac joint

## Abstract

Deep learning (DL) techniques have demonstrated remarkable capabilities in recognizing complex patterns in medical imaging data. In recent years, DL has been increasingly applied in clinical medicine for disease diagnosis and progression prediction. This study aimed to develop and validate a DL model for detecting sacroiliitis using pelvic anteroposterior (AP) radiographs. We retrospectively analyzed 1853 patients with pelvic AP radiographs, including 3706 sacroiliac joints (SIJs). Pelvic AP radiographs served as input data for the DL model development, while the presence or absence of sacroiliitis confirmed by pelvic computed tomography (CT) was used as the reference standard output data. Based on CT findings, 1463 of 1853 right SIJs showed evidence of sacroiliitis, while 390 had no sacroiliitis. Similar findings were observed in the left SIJs. The dataset was split with 70% (1297 images) for training and 30% (556 images) for validation. The areas under the curve (AUC) for our DL model on the validation dataset were 0.871 (95% confidence interval (CI): 0.834–0.907) and 0.869 (95% CI: 0.834–0.907) for the left and right SIJs, respectively. Diagnostic accuracies for sacroiliitis on the left and right sides were 85.4% and 86.3%, respectively. These results demonstrate that a DL model trained on pelvic AP radiographs with CT-confirmed diagnoses can effectively aid in the diagnosis of sacroiliitis.

## 1. Introduction

Sacroiliitis, an inflammation of the sacroiliac joint (SIJ), is a clinical feature observed in various rheumatic and nonrheumatic disorders, including ankylosing spondylitis, other spondyloarthropathies, Bechet’s disease, osteoarthritis, and infectious conditions [1]. Due to the nonspecific nature of clinical symptoms associated with sacroiliitis, diagnosis heavily relies on imaging studies [2]. In routine clinical practice, pelvic radiography is typically the initial imaging modality employed for diagnosing sacroiliitis. However, radiographic changes in the SIJ of patients with sacroiliitis often lack sensitivity and specificity, particularly during early disease stages [1,2,3]. This diagnostic limitation has been partially addressed through more sensitive imaging techniques such as computed tomography (CT) and magnetic resonance imaging (MRI) [1]. While MRI excels at detecting bone marrow edema as an indicator of sacroiliac joint inflammation, CT provides superior visualization of erosions, bone sclerosis, and ankyloses, and offers guidance for interventional procedures [4,5]. In clinical practice, CT is frequently preferred for sacroiliitis diagnosis due to its relatively lower cost compared to MRI. However, the high radiation exposure and limited accessibility of CT often restricts its use, making pelvic radiographs the most feasible option for initial diagnosis. Unfortunately, the low sensitivity of conventional radiography for detecting sacroiliitis frequently leads to missed diagnosis in clinical settings [2].

Machine learning (ML) encompasses computer algorithms capable of autonomous learning from data without explicit programming [6,7,8,9]. ML has effectively addressed limitations inherent in conventional image analysis methods, facilitating significant advancements in medical imaging [6,7,8,9]. Deep learning (DL), an advanced iteration of ML, integrates numerous hidden layers to construct artificial neural networks that mimic the structure and functions of the human brain. DL methodologies surpass conventional ML approaches, particularly in learning from unstructured and perceptual image data [10,11]. Importantly, DL can recognize image characteristics that may be imperceptible to human observers [10,11]. Among DL architectures, the convolutional neural network (CNN) has proven exceptionally effective for image recognition and classification tasks [12].

Recent research has explored DL applications for sacroiliitis diagnosis. In 2025, Uzelaltinbulat et al. developed a simplified custom CNN model using four MRI sequences and compared its performance with three state-of-the-art pretrained models [13]. Their proposed model achieved high diagnostic accuracy (0.977 for image-based and 0.951 for patient-based classification), demonstrating potential for clinical implementation. Similarly, Martins et al. introduced a DL approach using both 2D and 3D convolutional neural networks for sacroiliitis detection from MRI scans, with the 3D CNN yielding superior performance (66.6% validation accuracy) compared to 2D models [14].

The present study aimed to develop a CNN model using pelvic radiographs as input data to determine the presence of sacroiliitis. Additionally, we utilized the presence or absence of sacroiliitis confirmed by pelvic CT as the reference standard output data for model development and validation.

## 2. Materials and Methods

### 2.1. Subjects

We retrospectively recruited 1853 patients (3706 SIJs; mean age = 37.69 (standard deviation = ±10.87) years; male/female = 1254:599) who visited Ulsan University Hospital between January 2010 and December 2022. Inclusion criteria were (1) adults over 18 years of age; (2) patients with no history of bony fracture involving the SIJ, such as pelvic fracture; and (3) patients who underwent both pelvic anteroposterior (AP) radiograph and CT examinations on the same day. Exclusion criteria comprised (1) patients who underwent either a pelvic AP radiograph or a CT scan alone and (2) patients with a history of pelvic surgery. Based on CT evaluation, 390 SIJs were classified as without sacroiliitis, and 1463 SIJs with sacroiliitis among the 1853 right and left SIJs that were assessed.

### 2.2. Pelvic AP Radiograph and CT for SIJ Evaluation

All 1853 patients underwent both pelvic AP radiography and pelvic CT. The entire pelvis was scanned using a multidetector-row helical CT scanner (LightSpeed VCT, GE Healthcare, Milwaukee, USA), with images obtained while patients maintained a supine position on the CT table. CT acquisition parameters included a slice thickness of 0.625 mm and a tube voltage of 120 kV. A radiologist with over 10 years of experience in musculoskeletal imaging reviewed the pelvic AP radiographs and CT images of all included patients. The presence of sacroiliitis was confirmed based on findings from pelvic CT images, which served as the reference standard.

### 2.3. The Deep Learning Model

We developed a DL model using Python 3.8.8, TensorFlow 2.10.1 (with Keras), and the Scikit-Learn 1.1.2 library to diagnose sacroiliitis in the left and right SIJs from pelvic AP radiographs. We employed two pretrained CNN models based on EfficientNetV2S architecture and trained them from scratch on the left and right SIJ regions of interest (ROIs). Table 1 summarizes the architectural details of the employed model.

**Figure 1:**
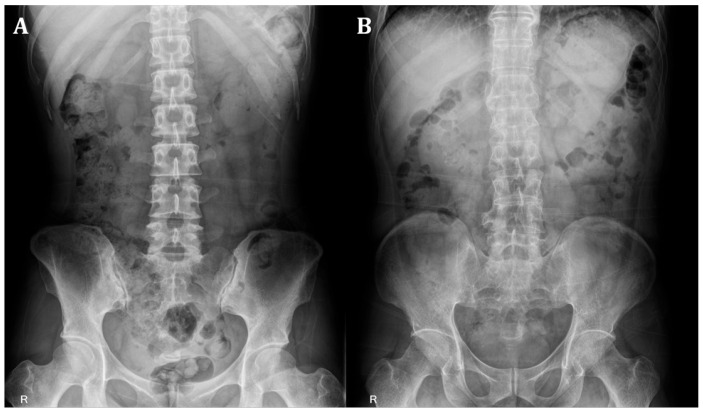
(**A**) Absence of sacroiliitis on bilateral sacroiliac joints. (**B**) Presence of sacroiliitis on bilateral sacroiliac joints.

From each patient’s pelvic AP radiograph, initial ROI images were extracted and standardized to a size of 600 (height) × 300 (width) pixels. The ROI images included the left and right SIJs. Two separate ROI images containing the left or right SIJ portions were extracted from the initial ROI images of size 2865 (height) × 2344 (width) pixels. These left and right SIJ ROI images were used for training the models to detect sacroiliitis. Figure 1 provides representative examples of absence and presence sacroiliitis on bilateral SIJs.

**Figure 2:**
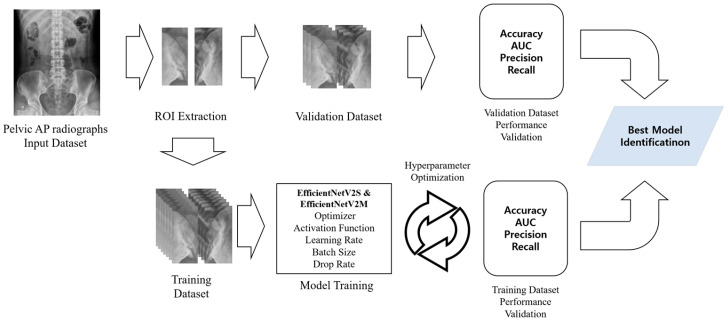
Flowchart illustrating the deep learning process for detecting sacroiliitis in pelvic anteroposterior radiographs, from image acquisition through anteroposterior radiographs and from image acquisition through region of interest extraction to final classification. Abbreviations: AP—anteroposterior; ROI—region of interest; AUC—area under the curve.

Figure 2 illustrates the overall process of sacroiliitis diagnosis using the proposed DL models, both designed for binary classification. In the models for diagnosing left and right sacroiliitis, each SIJs was classified as either 0 (absence of sacroiliitis) or 1 (presence of sacroiliitis). Results from the two models were then combined to determine the diagnosis for each patient, classified as either absence of sacroiliitis (left and right SIJs: 0), left sacroiliitis (left SIJ: 1, right SIJ: 0), right sacroiliitis (left SIJ: 0, right SIJ: 1), or bilateral sacroiliitis (left and right SIJs: 1).

**Figure 3:**
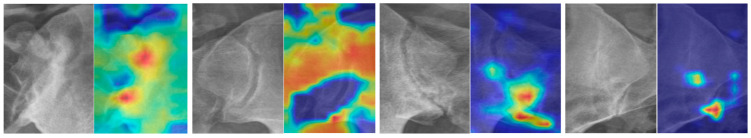
Region of interest and heatmap images. The heatmap colors delineate the intensity of model attention or activation within the region of interest. Warmer colors (red and orange) highlight areas of high importance that significantly influenced the model’s prediction. Cooler colors, (blue and purple) indicate regions of lower importance with minimal impact on the outcome.

The images in Figure 3 show the regions of interest and corresponding heatmaps. This representation effectively highlights variations in data intensity across the regions of interest, facilitating an intuitive visual assessment. The heatmap colors delineate the intensity of model attention or activation within the region of interest. Warmer colors, such as red and orange, highlight areas of high importance that significantly influenced the model’s prediction. In contrast, cooler colors, like blue and purple, indicate regions of lower importance with minimal impact on the outcome.

Table 1 shows the core architecture table [15] of the EfficientNetV2S model, and Table 2 summarizes the architectural details of the employed model.

### 2.4. Experiment

To mitigate overfitting risk in the developed model, the 1853 images were partitioned into two separate sets: 70% (1297 images) were assigned for training, and 30% (556 images) were allocated for validation. The specifics of the dataset configuration are summarized in Table 3. The ML model was trained using TensorFlow version 2.10.1 (Google, Mountain View, CA, USA) and the Scikit-Learn toolkit version 1.1.2.

### 2.5. Statistical Analysis

Statistical analyses were conducted using Python 3.8.10 and the Scikit-Learn toolkit version 1.1.2. Receiver operating characteristic (ROC) curve analysis was performed, and the area under the curve (AUC) was calculated. The 95% confidence interval (CI) for the AUC was determined using the method previously described by DeLong et al. [16].

## 3. Results

**Figure 4:**
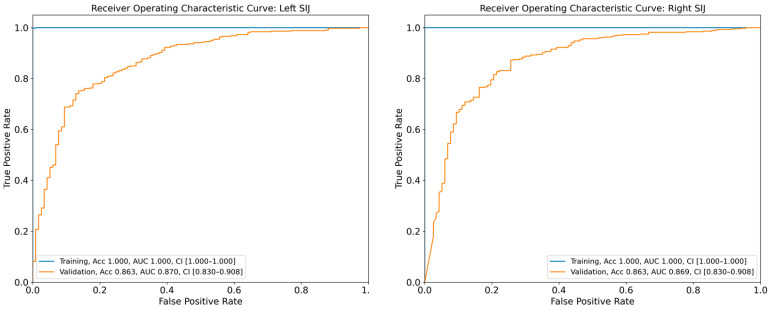
Receiver operating characteristic curve for the validation and test dataset of the anteroposterior pelvic radiographs for the deep learning model for diagnosing sacroiliitis. SIJ—sacroiliac joint; Acc—accuracy; AUC—area under the curve; CI—confidence interval.

The DL model achieved AUCs of 0.870 (95% CI: 0.830–0.908) and 0.869 (95% CI: 0.830–0.908) for the diagnosis of sacroiliitis in the left and right SIJs, respectively, based on the validation dataset (Figure 4). The diagnostic accuracies of sacroiliitis for the left and right sides were 86.3% and 86.3%, respectively, as assessed using the validation set. Additional performance metrics for the diagnostic models are presented in Table 3.

**Figure 5:**
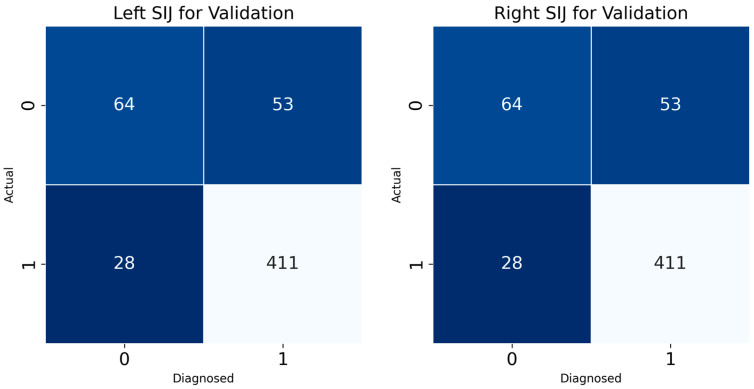
Confusion matrix for our developed models (tested with the validation dataset) (0—absence of sacroiliitis; 1—presence of sacroiliitis). SIJ—sacroiliac joint.

Confusion matrix results for the left and right SIJ models provided insight into the classification accuracy of the developed DL model for sacroiliitis diagnosis (Figure 5). For the left SIJ model validation dataset, of 117 “absence of sacroiliitis” images, 64 were correctly classified, while 28 were misclassified as “presence of sacroiliitis”. Of the 439 “presence of sacroiliitis” images, 411 were correctly classified, while 53 were misclassified as “absence of sacroiliitis”. Similarly, for the SIJ right model, of the 117 “absence of sacroiliitis” images, 65 were correctly classified, while 23 were misclassified as “presence of sacroiliitis”. Of the 439 “presence of sacroiliitis” images, 416 were correctly classified, while 54 were misclassified as “absence of sacroiliitis”.

For the purpose of performance comparison with the deep learning model developed in this study, a traditional machine learning technique, Support Vector Machine (SVM), was trained, and its performance was subsequently measured using validation data. The SVM employed a radial basis function (RBF) kernel, and the optimal model was selected after performing iterative training by adjusting the C parameter ten times.

The performance of the SVM designated as the ‘left model’ was as follows: accuracy of 0.787, precision of 0.815, recall of 0.942, and an F1-score of 0.873. For the ‘right model’, the performance metrics were an accuracy of 0.787, precision of 0.815, recall of 0.945, and an F1-score of 0.875. These results demonstrated a relatively lower performance for the SVM models compared to the deep learning model.

To benchmark the developed model’s diagnostic accuracy, two experienced clinicians (A and B) with over 15 years of experience in musculoskeletal imaging independently evaluated the presence of sacroiliitis using the same 556 pelvic AP radiographs from the validation set without access to CT images or DL model results. For the right and left SIJs, the diagnostic accuracies of clinicians A and B were 58.5% and 58.1%, and 57.4% and 58.8%, respectively.

## 4. Discussion

In this study, we developed a CNN model capable of determining the presence of sacroiliitis based on pelvic radiographs. Our model classified the output into “absence of sacroiliitis” and “presence of sacroiliitis”. The AUC values of our model, as evaluated on the validation dataset, were 0.871 and 0.869 for the left and right SIJs, respectively. Considering that an AUC between 0.8 and 0.9 is generally regarded as indicating excellent diagnostic capability, our model demonstrates highly effective performance for determining the presence of sacroiliitis using pelvic AP radiographs [17]. Furthermore, the diagnostic accuracy of our developed model (approximately 85%) substantially exceeded that of clinicians experienced in the field of musculoskeletal disorders.

A DL algorithm is constructed with a multilayer perceptron comprising multiple hidden layers and a feedforward neural network, enabling the recognition of detailed features or patterns within input data [18,19]. CNNs specifically process image data through repeated convoluting and pooling operations [20,21]. Through these processes, important features or patterns in the input data can be extracted and classified [19,20]. We postulate that our CNN algorithm can recognize valuable radiographic features and extract specific characteristics associated with sacroiliitis, such as erosion, subchondral sclerosis, change in joint space, and ankylosis, from the input images.

To our knowledge, two previous studies have developed DL models for detecting sacroiliitis in pelvic radiographs [22,23]. In 2021, Brassem et al. utilized 1553 pelvic AP radiographs (training: 1324, validation: 229) [22] and reported an AUC of 0.97, with sensitivity and specificity of 88% and 95%, respectively. In 2023, Üreten et al. analyzed 585 pelvic AP radiographs, with 70%, 15%, and 15% allocated for training, validation, and testing, respectively [23]. Their study employed VGG-16, ResNet-101, and Inception-v3 networks, achieving AUC values of 0.9–0.96 and accuracies of 82–89.9%.

While the diagnostic accuracies reported in these previous studies appear slightly higher than those of our model, it is crucial to note that their reference standard for sacroiliitis was based solely on radiographic findings, not on CT or MRI confirmation. The diagnosis of sacroiliitis using radiographs alone is limited. In 2017, Melchior et al. reported that the sensitivity and specificity of pelvic radiography for diagnosing sacroiliitis typically ranged between 70% and 80%. Moreover, in our study, the diagnostic accuracy based on pelvic radiographs was approximately 58% [3]. Therefore, the diagnostic performance of previously developed DL models may not reflect true accuracy against a more definitive reference standard.

Although CT is not as accurate as MRI for detecting the presence of sacroiliitis, its accuracy is significantly higher than that of radiography [2,3]. A distinctive strength of our study is the development of a DL model for detecting sacroiliitis based on pelvic radiographs, using CT-confirmed sacroiliitis status as the reference standard. This approach likely enables our model to more accurately represent the actual condition of the SIJ compared with previously developed models that relied on radiographic findings alone.

## 5. Conclusions

This study demonstrates that a DL model trained using AP pelvic radiographs can effectively assist in diagnosing sacroiliitis when validated against CT findings. Our model is unique because we used information on the presence or absence of sacroiliitis presented on pelvic CT as the output data. We believe this DL model could provide valuable clinical assistance for more accurate sacroiliitis diagnosis in situations where MRI or CT imaging is unavailable and only radiography can be performed.

However, our study has several limitations. First, we did not use pelvic MRI findings, which represent the gold standard for sacroiliitis diagnosis, to develop our DL model. Second, the relatively limited dataset size may constrain the model’s generalizability. We anticipate that integrating novel models, such as state space models [24,25,26], with DL techniques could address certain limitations of current research in this domain, particularly by enhancing temporal dynamics modeling and improving predictive robustness, thus warranting further investigation to overcome these limitations.

## Figures and Tables

**Table 1:** EfficientNetV2S architecture [15].

Stage	Operator	Stride	Number of Channels	Number of Layers
0	Conv3 × 3	2	24	1
1	Fused-MBConv1, k3 × 3	1	24	2
2	Fused-MBConv4, k3 × 3	2	48	4
3	Fused-MBConv4, k3 × 3	2	64	4
4	MBConv4, k3 × 3, SE0.25	2	128	6
5	MBConv6, k3 × 3, SE0.25	1	160	9
6	MBConv6, k3 × 3, SE0.25	2	256	15
7	Conv1 × 1 and Pooling and FC	-	1280	1

**Table 2:** Layer types and parameters in the model for diagnosing sacroiliitis on the pelvic anteroposterior radiograph.

Layer (Type)	Output Shape	Parameters
EfficientNetV2S	19 × 10 × 1280	327,680
GlobalAveragePooling2D	1280	0
BatchNormalization	1280	5120
Dropout	1280	0
Dense	1024	1,311,744
Dropout	1024	0
Dense	1	1025
Total params: 21,649,249		
Trainable params: 21,492,817		
Nontrainable params: 156,432		

**Table 3:** Performance analysis of the models for diagnosing sacroiliitis in the left and right sacroiliac joints.

	Left Sacroiliitis Model					Right Sacroiliitis Model				
**Sample size** **(patients)** **Sample ratio** **(patients)**	1297, 70% for training, 556, 30% for validation, total 1853 Absence of sacroiliitis: 390, 21.0%. Presence of sacroiliitis: 1463, 79.0% Absence of sacroiliitis: 273, 21.0%. Presence of sacroiliitis: 1024, 79.0% for training Absence of sacroiliitis: 117, 21.0%. Presence of sacroiliitis: 439, 79.0% for validation					1297, 70% for training, 556, 30% for validation, total 1853 Absence of sacroiliitis: 390, 21.0%. Presence of sacroiliitis: 1463, 79.0% Absence of sacroiliitis: 273, 21.0%. Presence of sacroiliitis: 1024, 79.0% for training Absence of sacroiliitis: 117, 21.0%. Presence of sacroiliitis: 439, 79.0% for validation				
**Model details**	- EfficientNetV2S CNN model with full training - Adam optimizer, ReLU activation - Learning rate 5 × 10^−5^, batch size 16, dropout rate 0.5 - Batch Normalization and dropout for regularization - ROI image resized to (600 × 300) and normalized - Training accuracy: 99.6%, AUC 1.000 with 95% CI [1.000–1.000] - Validation accuracy: 86.3%, AUC 0.871 with 95% CI [0.830–0.908]					- EfficientNetV2S CNN model with full training - AdamW optimizer, ReLU activation - Learning rate 5 × 10^−4^, batch size 8, dropout rate 0.2 - Batch Normalization and dropout for regularization - ROI image resized to (600 × 300) and normalized - Training accuracy: 100.0%, AUC 1.000 with 95% CI [1.000–1.000] - Validation accuracy: 86.3%, AUC 0.869 with 95% CI [0.830–0.908]				
**Model performance** **(validation data)**	Class	Precision	Recall	F1-score	Support	Class	Precision	Recall	F1-score	Support
	Absence of sacroiliitis (0)	0.696	0.547	0.612	117	Absence of sacroiliitis (0)	0.739	0.556	0.634	117
	Presence of sacroiliitis (1)	0.886	0.936	0.910	439	Presence of sacroiliitis (1)	0.889	0.948	0.917	439
	Weighted average	0.846	0.854	0.848	556	Weighted average	0.853	0.862	0.854	556

CNN—convolutional neural network; Adam—adaptive moment estimation; AdamW—adaptive moment estimation with weight decay; ReLU—rectified linear units; ROI—region of interest; ROC—receiver operating characteristics; AUC—area under the curve; CI—confidence interval.

## Data Availability

The datasets generated during and/or analyzed during the current study are available from the corresponding author on reasonable request.

## Abbreviations

AUC	area under the curve
CI	confidence interval
CNN	convolutional neural network
CT	computed tomography
DL	deep learning
ML	machine learning
MRI	magnetic resonance imaging
ReLU	rectified linear unit
ROI	region of interest
ROC	receiver operating characteristic
SIJ	sacroiliac joint
